# Supplementary material for: The impact of coal miners’ emotions on unsafe behavior: A study on the mediated effects with a moderating role
Source: PLoS One. 2025 Sep 22;20(9):e0331127. doi: 10.1371/journal.pone.0331127 (PMC12453193; doi:10.1371/journal.pone.0331127)
Supplement: Attachment — Submitted filename: PONE-D-25-18288.pdf [file pone.0331127.s002.pdf]

Thank you for giving me this opportunity to review，The theoretical model of this paper is innovative. It is the first time to integrate the dual dimensions of positive and negative emotions, the mediating mechanism of unsafe psychology and the moderating role of safety climate in the study of Miners' safety behavior, a multi-level theoretical framework is constructed.However, there are also the following problems: 1) the sample representation and method limitations, the sample size of the data is a bit small, the universality of the conclusion is questionable, and the data presentation and analysis results need to be further modified, the direction of effect of health status on unsafe behaviors was inconsistent in table 3(M1: $\beta = 0.166$; M4: $\beta = -0.011$) , please explain further. The text mentions Fig. 2, but the manuscript does not provide an illustration, which affects the interpretation of the results.
